# Peroxisome Proliferator-Activated Receptor-gamma agonists exhibit anti-inflammatory and antiviral effects in an EcoHIV mouse model

**DOI:** 10.1038/s41598-019-45878-6

**Published:** 2019-07-01

**Authors:** Amila Omeragic, Nareg Kara-Yacoubian, Jennifer Kelschenbach, Cigdem Sahin, Carolyn L. Cummins, David J. Volsky, Reina Bendayan

**Affiliations:** 1 Department of Pharmaceutical Sciences, Leslie Dan Faculty of Pharmacy, University of Toronto, Ontario, Canada; 2 Department of Medicine – Division of Infectious Diseases, Icahn School of Medicine at Mount Sinai, New York City, USA

**Keywords:** Neuroscience, HIV infections

## Abstract

The widespread use of combination antiretroviral therapy (cART) has resulted in significantly reduced deaths from HIV-1 associated complications and opportunistic infections. However, it is estimated that up to 50% of HIV-1 infected individuals still develop HIV-1 associated neurocognitive disorders (HAND). With no treatment currently available for patients, there is a critical need to identify therapeutic approaches that can treat this disorder. Evidence suggests that targeting Peroxisome Proliferator-Activated Receptor-gamma (PPARγ) can be anti-inflammatory in neurological disorders. Here we show that treatment with PPARγ agonists (rosiglitazone or pioglitazone) in primary cultures of mouse glial cells reversed EcoHIV-induced inflammatory genes (TNFα, IL-1β, CCL2, CCL3, CXCL10) and indicator of oxidative stress (iNOS). Furthermore, *in vivo*, mice administered with EcoHIV through intracranial injection resulted in upregulation of inflammatory genes (TNFα, IL-1β, IFNγ, CCL2, CCL3, CXCL10) and oxidative stress marker (iNOS) in the brain which was reversed through intraperitoneal administration of PPARγ agonists (rosiglitazone or pioglitazone). Finally, we demonstrated that treatment with these compounds *in vivo* reduced EcoHIV p24 protein burden in the brain. Our results suggest that treatment with PPARγ agonists are anti-inflammatory and antiviral in an *in vivo* model of EcoHIV infection. These drugs hold promise as potential candidates for HAND treatment in the future.

## Introduction

HIV-1 infection has been a prominent target for investigation and treatment, and many successful advances over the last two decades have greatly improved patient outcomes for those infected with the virus. The development and widespread use of combination antiretroviral therapy (cART) has resulted in significantly reduced deaths from HIV-1 associated complications and opportunistic infections^1,2^. However, despite the overall reduction of its incidence and severity, it is estimated that up to 50% of HIV-1 infected individuals still develop HIV-1 associated neurocognitive disorders (HAND)^3–5^. These can manifest as memory, motor, and/or behavioural deficits, at varying severities, which can affect quality of daily life and mortality rates in these patients^2,6^, yet the mechanisms contributing to HAND remain poorly understood. As a result, there is a lack of effective treatment for the condition, with patients relying on cART as the primary method of delaying or preventing the onset of cognitive impairment. This has proven to be insufficient in addressing the problem as several antiretroviral drugs (ARVs) exhibit poor blood-brain-barrier (BBB) permeability^7^ or have been linked with neurotoxicity (e.g. efavirenz)^8^. Sub-therapeutic concentrations of ARVs in the brain can result in low level virus replication, allowing the development of an HIV-1 sanctuary in the brain^7^. Thus, there is a need to identify other therapeutic approaches that can be effective for the prevention and treatment of HIV-associated brain inflammation and neurocognitive disorders.

HIV-1 can enter the brain very early on in the course of infection, and once in the central nervous system (CNS), primarly targets mononuclear phagocytes (e.g. perivascular macrophages and brain resident microglial cells) and to a lesser degree astrocytes^9^. Macrophages chronically infected with HIV-1 are resistant to viral apoptosis and resistant to post-infection cART, suggesting that this cell type can survive infection and serve as an HIV-1 reservoir^10,11^. Upon infection, microglia, macrophages and astrocytes become activated and secrete several pro-inflammatory cytokines and chemokines [i.e., tumor necrosis factor-α (TNFα), interleukin-1β (IL-1β), interferon-γ (IFNγ), C-C motif chemokine ligand 2 (CCL2), C-X-C motif chemokine 10 (CXCL10)] and neurotoxins [i.e., arachidonic/quinolinic acid and metabolites, platelet activating factor, neurotoxic amines, reactive oxygen species (ROS), nitric oxide (NO) and glutamate]^3,4,9^. Although neurons do not appear to be directly infected with HIV-1, chronic exposure to inflammatory, neurotoxic and oxidative stress markers during infection can cause neuronal injury and death^12^. In addition, several studies have reported that shed or secreted HIV-1 viral proteins, such as envelope glycoprotein (gp120), transactivator of transcription (Tat) and viral protein R (Vpr) can contribute to the neuropathogenesis of HIV-1^13,14^. Several mechanisms have been proposed for viral protein associated neuronal apoptosis, such as interactions with neuronal chemokine receptors, excitotoxicity due to glutamate accumulation, caspase activation, loss of mitochondrial membrane potential, and DNA fragmentation^9,12^.

The EcoHIV mouse model can be used as a versatile tool to study anti-inflammatory and antiviral targets, as it recapitulates many neuropathological features observed in HAND patients^15^. This chimeric virus replaces the coding region for gp120, which is responsible for binding and entry to human cells, with gp80, which is from ecotropic murine leukemia virus, allowing for the infection of mouse cells^15^. Recently, it has been demonstrated that mice infected with this chimeric strain show stable pro-virus in T-cells and macrophages, mucosal transmission of virus, normal CD4:CD8 ratios, a partially functional immune system and neurocognitive impairment resembling HAND^16^. Several other studies have further characterized this model by showing that mice infected with EcoHIV exhibit BBB impairments^17^, low level inflammatory responses in the brain^18^, and nigral degeneration^19^. Furthermore, other groups have used this model to investigate efficacy of ARVs^16,20^ and neuroprotective compounds^21^. Therefore, this established mouse model of HIV-1 neuropathogenesis was selected for our studies to investigate the effect of potential anti-inflammatory compounds in the context of HIV-1 associated brain inflammation and infection.

With the persistence of HAND among the HIV-1 infected population, and the lack of effective therapy, it is critical to identify potential therapeutic targets. Peroxisome proliferator-activated receptor-γ (PPARγ) is a ligand activated transcription factor belonging to the nuclear receptor family for steroid, thyroid hormones and retinoids. PPARγ plays a major role in lipid and glucose homeostasis^22^. Two synthetic PPARγ agonists rosiglitazone and pioglitazone, belonging to a class of drugs known as, thiazolidinediones, are FDA approved for treatment of type 2 diabetes. Several studies have demonstrated that the use of these agonists are neuroprotective in various animal models of neurological diseases^23,24^. In the context of HIV-1, there is also evidence suggesting that PPARγ agonists can play a neuroprotective role^25–27^. A recent study from our group showed that treatment with PPARγ agonists rosiglitazone or pioglitazone reduced HIV-1 gp120-induced inflammatory responses *in vitro*, utilizing primary cultures of rat mixed glial cells as well as *in vivo* in an intracerebroventricular administered HIV-1 gp120 rat model^28^. Moreover, the use of PPARγ agonist rosiglitazone was shown to exhibit direct anti-HIV effects in different cell types such as Th1Th17 cells^29^ and monocyte-derived macrophages^26^. In the clinic, pioglitazone appears to hold promise for the treatment of HIV-1 associated lipodystrophy syndrome (HALS)^30,31^ and hepatic steatosis in HIV/HCV patients^32^. This agonist also appears to be a safer PPARγ ligand with lesser cardiovascular side effects, even demonstrating reduced incidence of stroke in patients with type 2 diabetes^33^ and merits further investigation as a possible treatment for HAND. To date, there are no studies addressing the neuroprotective potential of pioglitazone in the context of HIV-1 *in vivo*. Additionally, the studies investigating the antiviral properties of PPARγ agonists were demonstrated with either rosiglitazone or older generation thiazolidinediones such as ciglitazone or troglitazone^26,29,34^. Although these studies are promising, it not known whether pioglitazone treatment will lead to similar outcomes.

The goal of this study was to investigate the brain inflammatory and viral responses in the EcoHIV mouse model, and subsequent therapeutic potential with PPARγ agonists, rosiglitazone and pioglitazone. We assessed the expression of inflammatory markers *in vitro*, utilizing primary cultures of mouse mixed glial cells exposed to EcoHIV, and *in vivo*, in mice intracranially injected with EcoHIV.

## Results

### PPARγ agonists rosiglitazone and pioglitazone reverse EcoHIV mediated inflammatory responses *in vitro*, in primary cultures of mouse mixed glial cells

**Figure 1:**
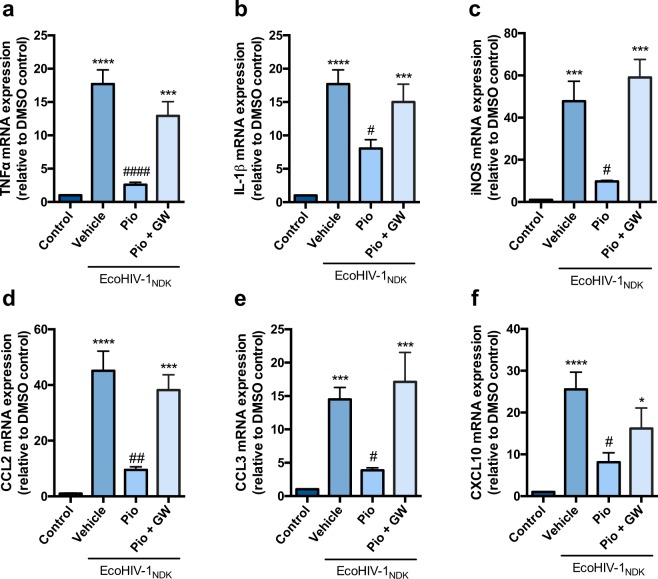
PPARγ agonist pioglitazone reverses EcoHIV-1 mediated inflammatory responses *in vitro*. Primary cultures of mixed mouse glial cells were treated with PPARγ agonist, pioglitazone (50 µM) or co-treated with PPARγ antagonist GW9662 (10 µM) for 1 h prior to EcoHIV-1 (17.5 ng of p24 per 1 × 10^6^ cells) exposure for 24 h. (**a**) TNF-α (**b**) IL-1β (**c**) iNOS (**d**) CCL2 (**e**) CCL3 and (**f**) CXCL10 mRNA levels were measured using qPCR. Cyclophilin B was used as the housekeeping gene. Results are expressed as mean ± SEM relative to the DMSO (control) of at least 3 separate experiments. Asterisks and pound symbol represent data points significantly different from DMSO (control) and EcoHIV-1 (vehicle) respectively (*p < 0.05, ***p < 0.001, ****p < 0.0001, ^#^p < 0.05, ^##^p < 0.01, ^####^p < 0.0001).

**Figure 2:**
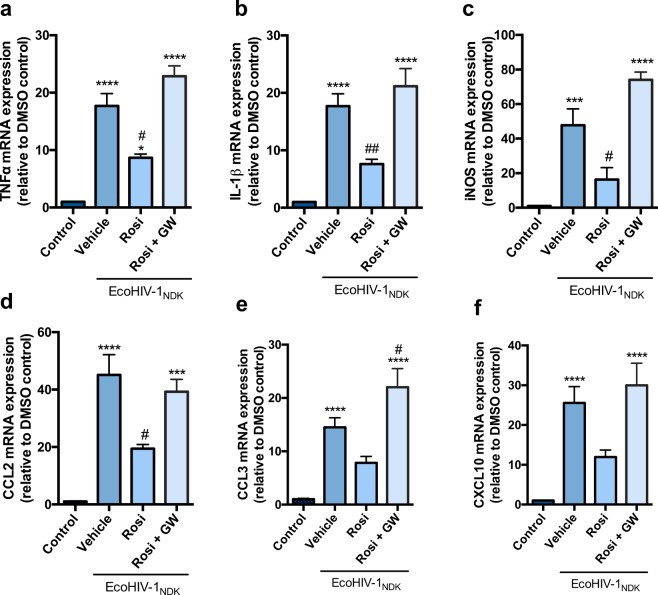
PPARγ agonist rosiglitazone reverses EcoHIV-1 mediated inflammatory responses *in vitro*. Primary cultures of mixed mouse glial cells were treated with PPARγ agonist, rosiglitazone (25 µM) or co-treated with PPARγ antagonist GW9662 (10 µM) for 1 h prior to EcoHIV-1 (17.5 ng of p24 per 1 × 10^6^ cells) exposure for 24 h. (**a**) TNF-α (**b**) IL-1β (**c**) iNOS (**d**) CCL2 (**e**) CCL3 and (**f**) CXCL10 mRNA levels were measured using qPCR. Cyclophilin B was used as the housekeeping gene. Results are expressed as mean ± SEM relative to the DMSO (control) of at least 3 separate experiments. Asterisks and pound symbol represent data points significantly different from DMSO (control) and EcoHIV-1 (vehicle) respectively (***p < 0.001, ****p < 0.0001, ^#^p < 0.05, ^##^p < 0.01).

We exposed mixed cultures of glial cells to EcoHIV (17.5 ng p24 per 1 × 10^6^ cells) for 24 h. This dosing regimen was based on previous studies using a comparable concentration in a similar *in vitro* system to induce an inflammatory response^35^. Exposure of the cells to EcoHIV significantly increased the inflammatory markers TNFα, IL-1β, CCL2, CCL3, and CXCL10; and an indicator of the oxidative stress response (iNOS) at 24 h post EcoHIV exposure (Figs 1 and 2). A heat-inactivated EcoHIV (HI-EcoHIV) control was performed in order to demonstrate specificity of the virus (Supplementary Fig. 1). Previous studies from our laboratory have shown that exposure to PPARγ agonists (rosiglitazone or pioglitazone) reversed HIV-1 gp120 induced mRNA expression of pro-inflammatory cytokines and oxidative stress markers in primary cultures of mixed rat astrocytes and microglia^28^. Herein, we confirm the anti-inflammatory effects of these PPARγ agonists in a robust *in vitro* model of HIV-1 associated inflammation. Treatment with pioglitazone (50 μM) or rosiglitazone (25 μM) reversed the inflammatory responses (Figs 1 and 2). Rosiglitazone treatment was not as effective as pioglitazone in reducing mRNA levels of chemokines CCL3 and CXCL10 (Fig. 2e,f). Other doses for pioglitazone (25 μM, 100 μM) and rosiglitazone (10 μM, 50 μM) were also examined for a dose response effect (Supplementary Figs 2 and 3). To confirm that the anti-inflammatory effects of PPARγ agonists rosiglitazone and pioglitazone were PPARγ dependent, cells were co-treated with the PPARγ specific antagonist, GW9662. As expected, we observed that GW9662 (10 μM) abolished the effects of both agonists (Figs 1 and 2). An additional control experiment was performed with GW9662 treatment alone in order to confirm that GW9662 was not inducing any inflammatory or toxic effects (Supplementary Figs 4 and 5). An MTT assay was also employed in primary cultures of mixed glial cells to verify that the treatments did not significantly alter cell proliferation and viability. In all conditions, cell viability was not significantly different from control (i.e., untreated) cultures (Supplementary Fig. 4).

### PPARγ agonists rosiglitazone and pioglitazone reverse EcoHIV mediated inflammatory responses *in vivo*, in a mouse model of EcoHIV infection

**Figure 3:**
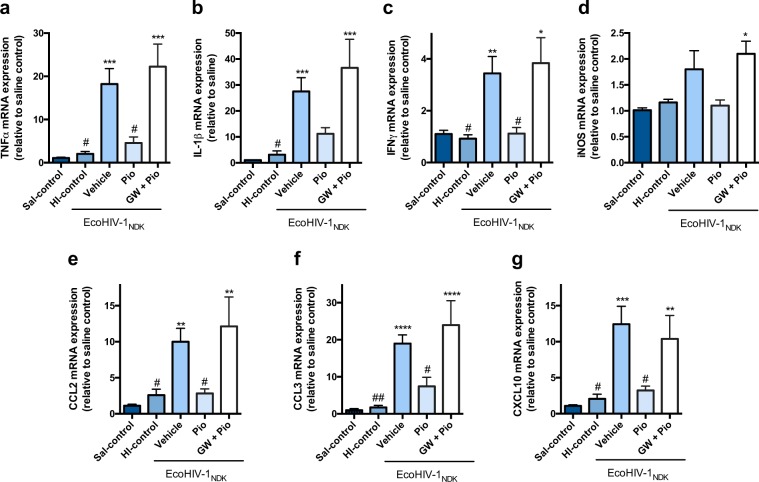
PPARγ agonist pioglitazone reverses EcoHIV-1 mediated inflammatory responses *in vivo*. Adult mice were administered IP, 30 min prior to IC unilateral injection of 1 × 10^6^ pg p24 of EcoHIV-1 with pioglitazone (20 mg/kg/day) or co-administration with GW9662 (5 mg/kg/day). An equal volume of HI- EcoHIV-1 was injected IC and this group was used as an additional control. The subcortical brain region was isolated 5 days post IC, and inflammatory markers (**a**) TNFα (**b**) IL-1β (**c**) IFNγ (**d**) iNOS (**e**) CCL2 (**f**) CCL3 and (**g**) CXCL10 mRNA levels were measured using qPCR. Cyclophilin B was used as the housekeeping gene. Results are expressed as mean ± SEM relative to saline group (control) n = 5–12 animals/group. Asterisks and pound symbol represent data points significantly different from saline (control) and EcoHIV-1 (vehicle) respectively (*p < 0.05, **p < 0.01, ***p < 0.001, ****p < 0.0001, ^#^p < 0.05, ^##^p < 0.01).

**Figure 4:**
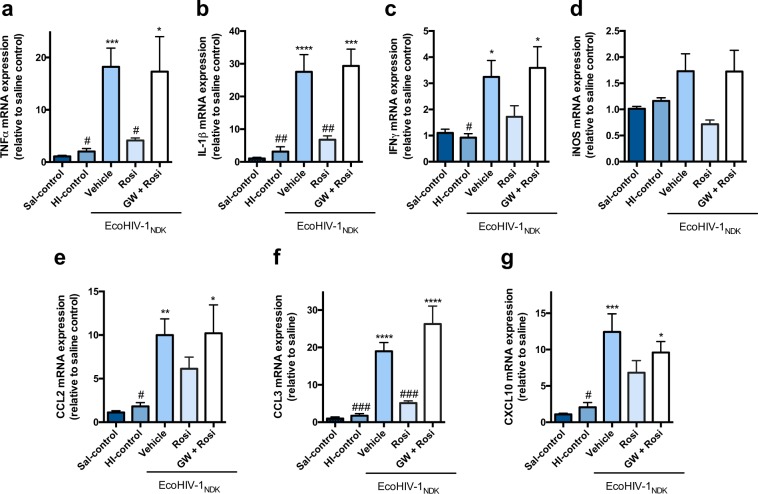
PPARγ agonist rosiglitazone reverses EcoHIV-1 mediated inflammatory responses *in vivo*. Adult C57BL/6 mice were administered IP, 30 min prior to IC unilateral injection of 1 × 10^6^ pg p24 of EcoHIV-1 with rosiglitazone (10 mg/kg/day) or co-administration with GW9662 (5 mg/kg/day). An equal volume of HI-EcoHIV-1 was injected IC and this group was used as an additional control. The subcortical brain region was isolated 5 days post IC, and inflammatory markers (**a**) TNFα (**b**) IL-1β (**c**) IFNγ (**d**) iNOS (**e**) CCL2 (**f**) CCL3 and (**g**) CXCL10 mRNA levels were measured using qPCR. Cyclophilin B was used as the housekeeping gene. Results are expressed as mean ± SEM relative to saline group (control) n = 5–12 animals/group. Asterisks and pound symbol represent data points significantly different from saline (control) and EcoHIV-1 (vehicle) respectively (*p < 0.05, **p < 0.01, ***p < 0.001, ****p < 0.0001, ^#^p < 0.05, ^##^p < 0.01, ^###^p < 0.001).

In our present study, the dose of EcoHIV for intracranial (IC) administration (1 × 10^6^ pg), was chosen based on previous reports using the same dose injected into the mouse brain to induce inflammatory and immune responses^18,36^. Herein, a single unilateral injection of EcoHIV (1 × 10^6^ pg) directly into the caudate putamen resulted in a significant increase in inflammatory markers TNFα, IL-1β, IFNγ, CCL2, CCL3 and CXCL10 5 days after infection (Figs 3 and 4). Although the levels of iNOS, appeared to be moderately elevated, these effects were modest and did not reach statistical significance (Figs 3d and 4d). To determine whether these effects were specifically mediated through EcoHIV infection, we peformed an additional control, injecting mice with HI-EcoHIV for the same time period. HI-EcoHIV failed to elicit an inflammatory response (Figs 3 and 4). To examine whether PPARγ agonists protect against EcoHIV-induced expression of inflammatory genes, mice infected with EcoHIV were treated with or without an IP injection of either 20 mg/kg pioglitazone or 10 mg/kg rosiglitazone daily for 5 days. Treatment with pioglitazone significantly reduced the levels of the following markers; TNFα, IFNγ, CCL2, CCL3 and CXCL10 (Fig. 3). Although a down regulatory trend was evident for IL-1β and iNOS, these markers did not reach statistical significance compared to the vehicle treated animals (Fig. 3b,d). Furthermore, treatment with rosiglitazone resulted in a significant downregulation of TNFα, IL-1β and CCL3 (Fig. 4); however, similar to the *in vitro* results, rosiglitazone was not as effective as pioglitazone and although a trend was present, it failed to significantly attenuate EcoHIV-induced expression of IFNγ, iNOS and the chemokines, CCL2 and CXCL10 (Fig. 4c,e,g). To verify that the anti-inflammatory effects of the PPARγ agonists were mediated through the PPARγ pathway, mice were co-administered with the PPARγ specific antagonist, GW9662 (5 mg/kg). As expected, co-treatment with GW9662 abolished the effects of both agonists (Figs 3 and 4).

### PPARγ agonists rosiglitazone and pioglitazone reduce EcoHIV viral gene and protein burden *in vivo*, in a mouse model of EcoHIV infection

**Figure 5:**
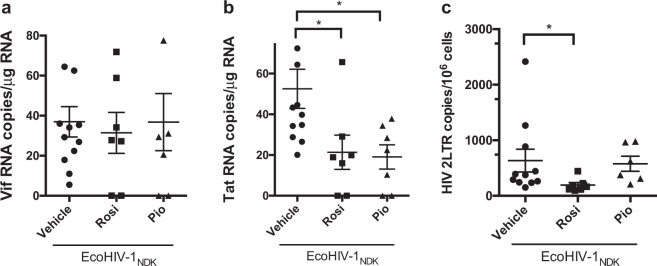
Viral gene burden in EcoHIV-1 infected mice. Adult C57BL/6 mice were administered IP, 30 min prior to IC unilateral injection of 1 × 10^6^ pg p24 of EcoHIV-1 with rosiglitazone (10 mg/kg/day) or pioglitazone (20 mg/kg/day). The subcortical brain region was isolated 5 days post inoculation and spliced viral genes (**a**) Vif (**b**) Tat and viral DNA (**c**) 2LTR were measured by qPCR. GAPDH was used as a housekeeping gene. Results are expressed as mean ± SEM; n = 5–12 animals/group. Asterisks symbol represents data points significantly different from EcoHIV-1 vehicle (*p < 0.05).

To confirm productive infection in mice, spliced HIV-1 viral genes (Vif and Tat) were quantified through qPCR as previously described^20^. Detection of spliced viral RNA was chosen because these viral forms have to be synthesized *de novo* from integrated provirus, and therefore represent new virus expression in the brain. Robust HIV-1 expression was found in brains of infected mice (Fig. 5a,b). Next, 2-LTR circular DNA was measured because unlike full-length DNA, this viral DNA form is not present in the HIV-1 inoculum and its detection by qPCR provides a quantitative measure of *de novo* HIV-1 infection. As expected, robust expression of 2-LTR DNA was seen in infected animals (Fig. 5C). The levels of viral RNA and DNA are comparable to those analyzed in brain tissue from mice infected with an equivalent dose and similar time frame^18,36^. Furthermore, the effect of each PPARγ agonist (pioglitazone or rosiglitazone) was examined in the context of reducing viral gene content. There were no differences in the levels of the Vif viral gene between vehicle and PPARγ agonist treated mice (Fig. 5a). However, the viral gene Tat, which plays a role in transcriptional regulation of the virus, was significantly downregulated in both pioglitazone and rosiglitazone treated groups (Fig. 5b) and 2LTR HIV DNA was significantly reduced in the rosiglitazone treated group (Fig. 5c).

**Figure 6:**
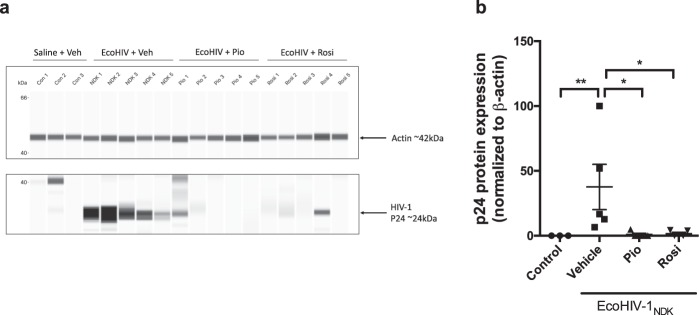
Viral protein burden in EcoHIV-1 infected mice. Adult C57BL/6 mice were administered IP, 30 min prior to IC unilateral injection of 1 × 10^6^ pg p24 of EcoHIV-1 with rosiglitazone (10 mg/kg/day) or pioglitazone (20 mg/kg/day). Cytoplasmic fractions of subcortical brain regions were isolated 5 days post inoculation and p24 protein expression was measured by Wes. β-actin was used as a loading control. (**a**) Representative blot which was cropped for clarity and (**b**) densitometric analysis. Full-length blots are presented in Supplementary Fig. 6S. Results are expressed as mean ± SEM; n = 3–5 animals/group. Asterisks symbol represents data points significantly different from EcoHIV-1 (vehicle) respectively (*p < 0.05, **p < 0.01).

To evaluate protein levels of the HIV-1 viral core protein, p24, mouse brain tissues from control, EcoHIV infected and EcoHIV injected with PPARγ treatment were subjected to immunoblotting (Fig. 6). As expected, p24 was undetected in control animals. Mice infected with EcoHIV showed robust but variable expression levels of p24 (Fig. 6a). Upon treatment with either rosiglitazone or pioglitazone, these levels were significantly decreased, and in most cases below the level of detection. These data indicate that both drugs exhibit antiviral effects.

## Discussion

The prevalence of HAND in the era of cART continues to be a health challenge with up to 50% of HIV-1 infected patients reported to be affected^3–5^. The underlying mechanisms for HAND still remain poorly understood; however, a major contributing factor is the chronic brain inflammatory response due to low level HIV-1 replication in viral reservoirs such as microglia^10^. Additionally, several reports suggest that shed or secreted viral proteins (e.g. gp120, Tat, Vpr) can also be neurotoxic^13,14^. Currently, there is no treatment available for HAND, and although the use of cART significantly extends the life span of HIV-1 infected patients, the effects of these drugs on improving neurocognitive performance are conflicting, and in certain instances, have been associated with neurotoxicity (e.g. efavirenz)^4,8^. The aim of the present study was to identify therapeutic compounds that can effectively exhibit anti-inflammatory properties without neurotoxicity.

Our group has previously demonstrated gp120-mediated inflammatory response *in vitro*, in primary cultures of rodent and human astrocytes as well as *in vivo*, in a rodent model of intracerebroventricular administered HIV-1_ADA_ gp120^28,37–40^. Although direct injection of recombinant gp120 protein results in a highly reproducible inflammatory response in cells and rodents, it presents several challenges and limitations for investigating treatment regimens and studying chronic HIV-related impairments. In the present study, we used a chimeric strain of EcoHIV which recapitulates many features of HAND, such as stable pro-virus in T-cells and macrophages, mucosal transmission of virus, normal CD4:CD8 ratio, a partially functional immune system and neurocognitive impairment^16^. This report, among several others supports the concept that this animal model can be used as a versatile tool for investigating potential anti-inflammatory and antiviral therapeutics^18,20,21^. Herein, we used primary cultures of mouse glial cells where exposure to EcoHIV resulted in a significant induction of a panel of inflammatory cytokines and chemokines (i.e. TNFα, IL-1β, IFNγ, iNOS, CCL2, CCL3 and CXCL10) which are reported to be implicated in the context of HAND^3,4^. As expected, the inflammatory response observed was significantly greater than previously reported with gp120 alone^28^, likely due to the presence of other viral proteins (e.g. Tat, Vpr) which are known to have detrimental effects in the brain^13,14^.

Next, we sought to investigate the anti-inflammatory potential of PPARγ agonists rosiglitazone or pioglitazone in reversing the upregulation of these markers. In addition to their widespread use for the treatment of type 2 diabetes, pioglitazone and rosiglitazone are known to exhibit neuroprotective effects in several models of CNS disorders^23,24^. To date, a few studies, including one from our group, have demonstrated the anti-inflammatory potential of PPARγ agonists in attenuating HIV-associated inflammatory response^25,28,41,42^. Aside from our previous study, reports are limited with regards to PPARγ agonist treatment in glial cells in the context of HIV-1. This is critical as microglia, and to a lesser extent astrocytes, are considered to be major targets of HIV-1, and infection of these cells significantly contributes to the neuropathogenesis of HIV^9^. Our *in vitro* data demonstrated that treatment of glial cells with rosiglitazone or pioglitazone reversed the EcoHIV-mediated inflammatory responses. Furthermore, we showed that co-treatment with GW9662, a synthetic PPARγ antagonist, abolished the rosiglitazone or pioglitazone anti-inflammatory effects, suggesting that these effects are specifically mediated by PPARγ.

Subsequent studies were aimed at characterizing an *in vivo* model of HIV-1 associated brain inflammation by IC administration of EcoHIV. A single unilateral injection of EcoHIV (1 × 10^6^ pg) directly into the caudate putamen resulted in a significant increase in inflammatory markers (TNFα, IL-1β, IFNγ, CCL2, CCL3, CXCL10) 5 days after infection (Figs 3 and 4). The caudate putamen, a region of the striatum, is one of the most affected brain regions during HIV-1 infection. High viral burden and abnormal pathology of this brain region have been reported^43,44^. We investigated the same markers as those selected for the *in vitro* work, and examined the anti-inflammatory properties of pioglitazone and rosiglitazone in this model. Our results showed that treatment with pioglitazone, administered IP daily for 5 days, significantly reduced expression levels of the markers; TNFα, IFNγ, CCL2, CCL3 and CXCL10 (Fig. 3). Furthermore, treatment with rosiglitazone, administered IP daily, also resulted in a significant downregulation of the markers TNFα, IL-1β and CCL3 (Fig. 4). Although a downregulation was evident for the other markers these did not reach statistical significance (Figs 3 and 4). It is interesting to note that rosiglitazone was not as effective as pioglitazone in reducing certain markers as observed from both the *in vitro* and *in vivo* results (Figs 2 and 4). Specifically, CCL2 and CXCL10, chemokines that play a critical role in signaling for recruitment of immune cells remained elevated *in vivo* (Fig. 4e,g). Upregulation of these markers can be especially detrimental during HIV-1 infection, as the recruitment of immune cells such as monocytes from the systemic circulation, can result in increased entry of HIV-1^45,46^. CXCL10 is associated with immune activation and general recruitment of immune cells^47^, whereas CCL2 induces a specific effect of recruiting HIV-1 infected leukocytes across the BBB^48^. The presence of increased CCL2 and CXCL10 in HIV-positive patients with neuroinflammation or neurocognitive deficits suggests that these markers play a critical role in neuropathogenesis of HAND^47,49–51^. It is possible that an increased dose of rosiglitazone would be more effective at reducing CCL2 *in vivo*, as we did observe a significant reduction of this chemokine *in vitro*, where the concentration was likely higher than what would be expected in the brain after a 10 mg/kg IP injection. Although both PPARγ agonists demonstrate anti-inflammatory benefits *in vitro* and *in vivo*, our data suggests that pioglitazone exhibits more potent anti-inflammatory effects and could be the preferred PPARγ agonist treatment option.

Finally, we investigated viral burden in infected and treated mice. To the best of our knowledge, this is the first study examining the antiviral potential of pioglitazone. P24 is an HIV viral core protein encoded by the HIV gag gene. In the clinical setting, detection of p24 antigen by either ELISA or western blot assays are common methods applied for assessing HIV-1 infection. It has previously been demonstrated that PPARγ agonist rosiglitazone can exhibit direct anti-HIV effects in different cell types such as Th1Th17 cells^29^, monocyte-derived macrophages^26^ and dendritic cells^34^. Additionally, this was also demonstrated *in vivo*, using a humanized mouse model of HIV-1 encephalitis where treatment with rosiglitazone reduced HIV-1 replication in the brain as demonstrated through reduced p24 content via immunohistochemical analysis^26^. Herein, we demonstrated reduced p24 protein content through western blot analysis. These data corroborate the findings from the previous study, however, for the first time we showed that the PPARγ agonist pioglitazone is also effective in reducing viral replication. Some of the mechanisms for reducing viral replication that have been proposed include reduction of HIV-1 LTR promoter^26,29^. For example, rosiglitazone has been reported to suppress the redox regulated transcription factor NF-κB. NF-κB binding sites have been identified in the promoter-proximal enhancer region of HIV-1 LTR^52^. Another important regulator of HIV replication is Tat, a viral protein which serves as an activator of HIV-1 transcription^53^. Tat presents a transcriptional activation domain composed of a cysteine rich region and a hydrophobic core motif, along with an arginine-rich RNA-binding motif that allows binding of Tat to the transactivator response element on the RNA structure on HIV-1^53^. Due to its prominent role in HIV-1 viral transcription, its downregulation by rosiglitazone and pioglitazone could be a potential mechanism for the reduced HIV-1 viral replication observed. Additionally, the anti-inflammatory environement established by pioglitazone and rosiglitazone could contribute to a lower viral load. The relationship between HIV-1 replication and inflammation presents a viscious cycle where, HIV persistence induces inflammation that in turn contributes to HIV replication. For example, upregulation of adhesion molecules during inflammatory states can promote virus-induced cell-cell transfer, subsequently speading the virus^54^. *In vitro* and *in vivo* studies have also identified specific cytokines as critical factors modulating immunological and virological mechanisms associated with promoting HIV persistence^55,56^.

As previously indicated, PPARγ is a key regulator of fat metabolism and therefore, targeting this nuclear receptor can be useful in metabolic disorders such as lipodystrophy, dyslipidaemia and insulin resistance, which are all commonly associated with cART^57^. These metabolic disorders are referred to as HIV/cART associated lipodystrophy syndrome (HALS). Studies have demonstrated that PPARγ expression is reduced in patients with HALS^30,58^. As a result, PPARγ agonists could be a promising therapeutic target not only for neurocognitive disorders associated with HIV-1, but also metabolic abnormalities. A few studies have already been conducted examining the therapeutic potential of these drugs in the context of HALS. One clinical trial showed that treatment with pioglitazone improved limb fat atrophy and lipid profile in cART treated individuals^30^. A meta-analysis assessing rosiglitazone or pioglitazone use in the context of HIV-1 lipoatrophy, reported that pioglitazone was more effective at increasing limb fat mass whereas, rosiglitazone did not achieve the same effects^59^. This evidence further supports the potential use of pioglitazone as a new therapy for HIV infected patients not only for neurological complications but also potentially for metabolic disorders experienced by those suffering from HIV.

In addition to increased effectiveness in reducing inflammatory responses and restoring metabolic function, pioglitazone is also thought to have a safer profile on cardiovascular toxicity profile in comparison to rosiglitazone^33,60^. For example, results from the PROactive study, 34 month randomized controlled trial with 5238 patients showed positive cardiovascular effects, specifically a 50% reduction of stroke events after 3 years of treatment^33^. In an another study, the use of pioglitazone was associated with significant lower risk of all-cause mortality in comparison to the standard type 2 diabetes medication, metformin^60^. Pioglitazone has a more favourable lipid profile which could explain its positive cardiovascular effects^33^.

Identifying effective compounds that can target chronic inflammation in the context of HAND is therapeutically important, since most of the ARVs do not exhibit anti-inflammatory effects, with the exception of maraviroc. The anti-inflammatory effects of rosiglitazone and pioglitazone on cytokine and chemokine levels are exciting and could prevent neurodegeneration and recruitment of immune cells to the CNS. Novel treatment options are desperately needed to prevent/treat HAND, as the high percentage of HIV-1 infected patients suffering from this comorbidity can in all likelihood cause a high socioeconomic burden. Our study demonstrates that both PPARγ agonists rosiglitazone and pioglitazone are able to successfully reduce inflammatory markers in EcoHIV infected mice. These compounds are known to penetrate the BBB^61,62^, and as our *in vitro* data demonstrate, can act on reducing inflammation in microglia and astrocytes, primary cellular targets of HIV-1 in the brain. Additionally, both compounds demonstrated antiviral effects, potentially mediated through downregulation of the HIV-1 Tat. Although a limitation to this study is that EcoHIV does not contain gp120, which our group and several others have shown induces neurotoxic effects; our previous study^28^ is complimentary to the work presented herein and collectively, provides evidence that these drugs are effective in the context of HIV-1 associated brain inflammation.

## Materials and Methods

### Plasmids, virus, infection

EcoHIV-1_NDK_ chimeric virus was prepared as previously described^20^ and referred to as EcoHIV throughout the manuscript. The full nucleotide sequence of EcoHIV is available on GenBank (NCBI-NIH) through accession number MG470653.1. In brief, virus stocks were generated with the transfection of EcoHIV-1_NDK_ plasmid DNA into HEK293T cells. Cultured media was concentrated and titered for p24 antigen content with the use of HIV p24 AlphaLISA® Detection Kit (Perkin Elmer). EcoHIV was inactivated by heating at 57 °C for 45 min. All viruses were inoculated through cell exposure in media *in vitro*, or IC injection *in vivo*.

### Cell isolation, culture, and treatment

Primary cultures of mouse mixed glial cells (microglia and astrocytes) were prepared as described previously^63^ with a few modifications. All procedures were approved by the University of Toronto Institutional Animal Care and Use Committee under the protocol #20012207. All procedures were carried out in accordance with the Canadian Council on Animal Care. In brief, whole brain isolates were extracted from neonatal C57BL/6 mice pups aged 1–2 days (Charles River Laboratories, Canada) via decapitation. Cerebral cortices were dissected and the meninges were removed. Cortices were incubated in serum-free medium containing 1 mg/mL DNase I (Roche Applied Science) and 25 mg/mL porcine pancreatic trypsin (Sigma Aldrich) for 30 min Brain tissues were then mechanically grinded using a strainer mesh apparatus to produce a mixed glial cell suspension. The suspension was centrifuged for 5 minutes at 200 g and resuspended in primary glial culture medium containing Dulbecco’s Modified Eagle’s Medium (Sigma Aldrich, D5796), 10% heat inactivated fetal bovine serum (ThermoFisher Scientific), and 1X Penicillin-Streptomycin (Wisent Inc.). Mixed glial cells from 4 brain cortices were plated onto 50 μg poly-D-lysine coated 75 cm^2^ polystyrene tissue culture flasks (Sarstedt) and incubated at 37 °C in 5% CO_2_/95% air for 7–10 days or until confluence was attained. Cells were split into 25-cm^2^ flasks at the appropriate concentrations 72 h prior to experiments.

All treatments were performed on monolayers of primary cultures of mixed mouse glial cells grown in 25-cm^2^ tissue culture flasks. At the beginning of each experiment, cultured cells were incubated with fresh medium exposed to EcoHIV (17.5 ng of p24 per 1 × 10^6^ cells) for 24 h. This concentration was selected based on previous reports using a similar concentration in cultured astrocytes and microglia^35^. We performed an MTT assay to confirm that there was no toxicity observed at this concentration (Supplementary Fig. 4). All experiments were conducted at 37 °C in 5% CO_2_/95% air. Stock solutions of pioglitazone, rosiglitazone (Cayman Chemicals) and GW9662 (Sigma Aldrich) were dissolved in DMSO. A total volume of 5 µL drug solution was added to 5 mL media in T-25 flasks, resulting in a final concentration of 0.1% DMSO. The same final concentration of DMSO was used between all treatment groups (e.g. control, vehicle EcoHIV). Cells were treated with PPARγ agonists pioglitazone (25–100 μM) or rosiglitazone (10–50 μM) 1 h prior to EcoHIV exposure for 24 h. GW9662 (10 μM) was added 30 min prior to either agonist. Cell suspensions were collected 24 h after EcoHIV exposure, and prepared for qPCR analysis as described below. Cell viability was assessed in primary cultures of mouse glial cells treated with EcoHIV using a standard MTT assay previously described by our laboratory^38^.

### Real-time quantitative polymerase chain reaction (qPCR)

Real-time quantitative Polymerase Chain Reaction (qPCR) was applied to determine the transcript levels of inflammatory and oxidative stress markers as previously described^28^. Briefly, total RNA was extracted from cell culture or brain tissue using TRizol reagent. The concentration of RNA was quantified spectrophotometrically by measuring absorbance at 260 nm. Extracted RNA (2000ng) was treated with amplification grade DNase I to remove contaminating genomic DNA. The high capacity cDNA reverse transcriptase kit was used to synthesize first-strand cDNA. Mouse primers were purchased from ThermoFisher Scientific for the following genes using TaqMan technology: TNFα (Mm00443258_m1), Il-1β (Mm00434228_m1), iNOS (Mm00440485_m1), IFNγ (Mm01168134), CCL2 (Mm00441243_m1), CCL3 (Mm00441259_m1), CXCL10 (Mm00445235_m1) and cyclophilin B (housekeeping gene; Mm00478295_m1). Expression levels were normalized to housekeeping gene, cyclophillin B and compared to saline-treated control group using the comparative Ct (ΔΔCt) method.

### Western blot

Brain tissue homogenates were prepared using an NE-PER nuclear and cytoplasmic extraction kit from ThermoFisher Scientific. The extracts were prepared as per the manufacturer’s protocol. A concentration of 1 mg/mL was found to be optimal for a p24 antibody tested on 12–230 kDa Wes Separation Module capillary cartridges of Simple Protein Wes system (ProteinSimple). The primary mouse monoclonal antibody specific for HIV-1 p24 antibody was a generous gift from Dr. Alan Cochrane, Department of Molecular Genetics, University of Toronto, Toronto, ON, Canada and was used at a dilution of 1:50. A primary mouse monoclonal antibody specific for Beta-actin (Santa Cruz, A1978) was used as a loading control at a dilution of 1:4000. Anti-mouse detection modules for Wes (ProteinSimple) kits include Luminol-S, Peroxide, antibody Diluent 2, Streptavidin-HRP and anti-rabbit secondary antibody. Sample proteins were allowed to separate by a capillary technology and were analyzed based on the chemiluminescence signal peaks generated, which were transformed into digital images depicting bands as observed in western blot analysis. Using Compass software (ProteinSimple), the peak areas of p24 proteins were estimated and normalized against Beta-actin. The peak areas are directly proportional to the amount of target protein. Protein abundance of p24 was detected from cytoplasmic extracts from mouse brain tissue.

### Measurement of EcoHIV burden

HIV-1 viral genes Vif and Tat standardized by glyceraldehyde 3-phosphatase dehydrogenase (GAPDH) housekeeping were conducted as previously described^20^. In brief, custom Taqman qPCR primers were purchased from Thermo Fisher Scientific and amplification was conducted in an Applied Biosystems 7500 instrument. To unambiguously detect newly synthesized EcoHIV RNA, primers were designed to amplify a region in Vif generated by splicing (forward primer, 5′-AAGAGGCGAGGGGCAGCGA-3′; reverse primer, 5′-TCTTTACTTTTCTTCTTGGTACTACCTTTATG-3′; probe, 5′(FAM)-AGTAGTAATACAAGACAATAGTG(MGBNFQ)-3′) and Tat forward primer ND-tat-F (5′-CCTAGGACTGCTTGTAATAAGTGT-3′), reverse primer ND-tat-R2 (5′-GTCGGGTCCCCTCGGGACTGGGAG-3′), and probe ND-tat-P (5′-(FAM)-AAAGGCTTAGGCATCTC-(MGBNFQ)-3′). For the detection of EcoHIV 2LTR circular DNA by qPCR, brain tissue was homogenized in Trizol, and DNA extracted bysequential mixing with DNAzol, ethanol precipitation and washing, and NaOH treatment. DNA was buffered and then employed for qPCR, the reaction mixture contained custom-designed forward primer 2LTRF3 (5′-CTAGAGATCCCTCAGATCCGTTTAGT-3′),reverse primer 2LTRR1 (5′-TGGTGTGTAGTTCTGCCAATCG-3′), and probe 2LTRP (5′-(FAM)-TTTGGGTCTACAACACACAAGGCATCTTCC-(MGBNFQ)-3′). A standard curve for quantitation of viral gene or DNA copy number was constructed using graded numbers of a plasmid containing Vif, Tat or LTR-LTR junction. Every qPCR assay of virus burden included cell extracts from uninfected mice as negative controls.

### Animals

C57BL/6J mice (male, 8 weeks) were purchased from Charles River Laboratories and were housed at the University of Toronto Division of Comparative Medicine with rodent chow and water on a 12 h light-dark cycle. All procedures were approved by the University of Toronto Institutional Animal Care and Use Committee under the protocol #20012008. All procedures were carried out in accordance with the Canadian Council on Animal Care. The mice were randomly assigned to one of the following groups: Control, HI-EcoHIV, EcoHIV, pioglitazone + EcoHIV, rosiglitazone + EcoHIV, pioglitazone/GW662+ EcoHIV and rosiglitazone/GW9662+ EcoHIV, each group n ≥ 5 except HI-EcoHIV (n = 4).

### Animal surgery and IC administration of EcoHIV

Sterile stereotaxic technique was performed for all mouse brain injections as previously described by our group^28^. In brief, 2–5% isoflurane was used to induce surgical anesthesia. Prior to IC, animals were administered subcutaneously ketoprofen (5 mg/kg) to induce analgesic effect. Anesthetized mice were administered a single unilateral IC injection of EcoHIV (1 × 10^6^ pg of p24 at a rate of 0.5 μL/minute) and sacrificed 5 days post injection. A 5 μL Hamilton syringe was used to inject unilaterally into the caudate putamen at the following coordinates 2.5 mm lateral from midline and 2.5 mm ventral from the surface of the skull. Control animals received an equal volume of saline or HI-EcoHIV. Five days following IC injection, animals were anaesthetized and perfused through the left ventricle of the heart with a 30 mL phosphate buffered saline (PBS). Subcortical brain regions from the same side of injection were collected and harvested for further molecular and biochemical analysis. Samples were flash frozen in liquid nitrogen and kept at −80 °C.

### Drugs and treatments

Prior to EcoHIV IC injection, mice (n ≥ 5 per group) were randomly selected for treatment with PPARγ agonists rosiglitazone (10 mg/kg/day) or pioglitazone (20 mg/kg/day) or co-treatment of either one of the agonists with PPARγ antagonist GW9662 (5 mg/kg/day), administered via IP injections 30 min prior to IC injection of EcoHIV and for the duration of the experiment, 5 days. The doses of PPARγ agonists and antagonists were chosen based on previous studies^26,28,64^. Both rosiglitazone and pioglitazone have been reported to cross the BBB^61,62^. IP formulations were prepared via dissolution of each compound in a final concentration of 5% DMSO and 5% Tween-80 in PBS. Mice receiving IC injection of saline (control), HI-EcoHIV and EcoHIV received equal IP administration of PBS solution.

### Data analysis

Data was analyzed using GraphPad Prism software. A p-value less than 0.05 was considered statistically significant. Comparisons between groups for the inflammatory gene data was performed using one-way ANOVA with Bonferroni’s post-hoc analysis. Comparisons between groups for the viral gene and protein data was performed using one-way ANOVA with Kruskal-Wallis analysis.

## Supplementary information

Supplementary Info

## Data Availability

The datasets used and or analyzed during the current study are available from the corresponding author on reasonable request.
