# Supplementary material for: Peroxisome Proliferator-Activated Receptor-gamma agonists exhibit anti-inflammatory and antiviral effects in an EcoHIV mouse model
Source: Sci Rep. 2019 Jul 1;9:9428. doi: 10.1038/s41598-019-45878-6 (PMC6603270; doi:10.1038/s41598-019-45878-6)
Supplement: Supplementary file 1 — Supplementary Info [file 41598_2019_45878_MOESM1_ESM.docx]

**Title Page**

Peroxisome Proliferator-Activated Receptor-gamma agonists exhibit anti-inflammatory and antiviral effects in an EcoHIV mouse model

Amila Omeragic^1^, Nareg Kara-Yacoubian^1^, Jennifer Kelschenbach^2^, Cigdem Sahin^1^, Carolyn Cummins^1^, David J. Volsky^2^ and Reina Bendayan^1*^

*^1^Department of Pharmaceutical Sciences, Leslie Dan Faculty of Pharmacy, University of Toronto, Ontario, Canada*

***^2^****Department of Medicine – Division of Infectious Diseases, Icahn School of Medicine at Mount Sinai, New York City, USA*

***Address correspondence to Reina Bendayan**

[**r.bendayan@utoronto.ca**](mailto:r.bendayan@utoronto.ca)

Reina Bendayan, Pharm. D.

Professor and Career Scientist, Ontario HIV Treatment Network, MHO

Department of Pharmaceutical Sciences

Leslie Dan Faculty of Pharmacy

University of Toronto

144 College Street

Toronto, ON

M5S 3M2

**Figure 1S.**  EcoHIV-1 mediated inflammatory responses *in vitro*. Primary cultures of mixed mouse glial cells were treated with EcoHIV-1 (17, 500pg of p24) exposure for 24 h. An equal amount of heat-inactivated EcoHIV control was used as an additional control. (A) TNF-α (B) IL-1β (C) iNOS (D) CCL2 (E) CCL3 and (F) CXCL10 mRNA levels were measured using qPCR. Cyclophilin B was used as the housekeeping gene. Results are expressed as mean ± SEM relative to the DMSO (control) of at least 3 separate experiments. Asterisks represent data points significantly different from DMSO (control) (****p < 0.0001).

**Figure 2S.** PPARγ agonist pioglitazone reverses EcoHIV-1 mediated inflammatory responses *in vitro*. Primary cultures of mixed mouse glial cells were treated with increasing concentrations of PPARγ agonist, pioglitazone (25 µM, 50 µM, 100µM) for 1 h prior to EcoHIV-1 (17, 500pg of p24) exposure for 24 h. (A) TNF-α (B) IL-1β (C) iNOS (D) CCL2 (E) CCL3 and (F) CXCL10 mRNA levels were measured using qPCR. Cyclophilin B was used as the housekeeping gene. Results are expressed as mean ± SEM relative to the DMSO (control) of at least 3 separate experiments. Asterisks and pound symbol represent data points significantly different from DMSO (control) and EcoHIV-1 (vehicle) respectively (***p < 0.001, ****p < 0.0001, #p < 0.05, ##p < 0.01, ###p < 0.001, #### p < 0.0001).

**Figure 3S.** PPARγ agonist rosiglitazone reverses EcoHIV-1 mediated inflammatory responses *in vitro*. Primary cultures of mixed mouse glial cells were treated with increasing concentrations of PPARγ agonist, rosiglitazone (10 µM, 25 µM, 50 µM) for 1 h prior to EcoHIV-1 (17, 500pg of p24) exposure for 24 h. (A) TNF-α (B) IL-1β (C) iNOS (D) CCL2 (E) CCL3 and (F) CXCL10 mRNA levels were measured using qPCR. Cyclophilin B was used as the housekeeping gene. Results are expressed as mean ± SEM relative to the DMSO (control) of at least 3 separate experiments. Asterisks and pound symbol represent data points significantly different from DMSO (control) and EcoHIV-1 (vehicle) respectively (***p < 0.001, ****p < 0.0001, #p < 0.05, ##p < 0.01, ###p < 0.001).

**Figure 4S.** Effect of DMSO, EcoHIV-1, PPARγ agonists and PPARγ antagonist on cell viability *in vitro*. Primary cultures of mixed mouse glial cells were treated with either DMSO, EcoHIV-1 and rosiglitazone, pioglitazone at the respective concentrations for 24 h and cell viability was assessed using MTT assay. Results are expressed as percent of control and reported as mean ± SEM of at least 3 separate experiments.

**Figure 5S.** PPARγ antagonist GW9662 treatment does not induce inflammatory responses *in vitro*. Primary cultures of mixed mouse glial cells were treated with 10 µM GW9662 for 24 h. (A) TNF-α (B) IL-1β (C) iNOS (D) CCL2 (E) CCL3 and (F) CXCL10 mRNA levels were measured using qPCR. Cyclophilin B was used as the housekeeping gene. Results are expressed as mean ± SEM relative to the DMSO (control) of at least 3 separate experiments.

**a b**

**Figure 6S.** Viral protein burden in EcoHIV-1 infected mice. Adult C57BL/6 mice were administered IP, 30 min prior to IC unilateral injection of 1x10^6^pg p24 of EcoHIV-1 with rosiglitazone (10 mg/kg/day) or pioglitazone (20 mg/kg/day). Cytoplasmic fractions of subcortical brain regions were isolated 5 days post inoculation and p24 protein expression was measured by Wes (A). β-actin was used as a loading control (B). Results are expressed as mean ± SEM; n=3-5 animals/group.
